# Modeling the arousal potential of epistemic emotions using Bayesian information gain: a framework for inquiry cycles driven by free energy fluctuations

**DOI:** 10.3389/fpsyg.2025.1438080

**Published:** 2025-05-13

**Authors:** Hideyoshi Yanagisawa, Shimon Honda

**Affiliations:** Graduate School of Engineering, The University of Tokyo, Tokyo, Japan

**Keywords:** emotion, free energy, Bayes, arousal, curiosity, interest, inquiry

## Abstract

Epistemic emotions, such as curiosity and interest, drive the inquiry process. This study proposes a novel formulation of these emotions using two types of information gain derived from the principle of free energy minimization: Kullback–Leibler divergence (KLD), representing free energy reduction through recognition, and Bayesian surprise (BS), representing free energy reduction via Bayesian updating. Conventional Gaussian models predict an infinite divergence in information gain (KLD and BS) as prediction error increases, which contradicts the known limits of human cognitive resources. The key novelty of this study lies in a simple yet impactful modification: incorporating a uniform distribution into the Gaussian likelihood function to model neural activity under conditions of large prediction error. This modification yields an inverted U-shaped relationship between prediction error and both KLD and BS, producing a finite peak in information gain that better reflects cognitive realism. Based on this convexity, we propose that alternating the maximization of BS and KLD generates an ideal inquiry cycle that fluctuates around an optimal arousal level, with curiosity and interest driving this process. We further analyze how prediction uncertainty (prior variance) and observation uncertainty (likelihood variance) affect the peak of information gain. The results suggest that greater prediction uncertainty (reflecting open-mindedness) and lower observation uncertainty (indicating focused observation) promote higher information gains through broader exploration. This mathematical framework integrates the brain's free energy principle with arousal potential theory, providing a unified explanation of the Wundt curve as an information gain function and proposing an ideal inquiry process driven by epistemic emotions.

## 1 Introduction

Inquiry is an essential cognitive process in human activities such as scientific research, creation, and education. American philosopher Charles Sanders Peirce defines inquiry as a cycle of three inferences: abduction, deduction, and induction (Peirce, 1974). In the observation of surprising phenomena, abduction infers a possible cause of the observation, deduction predicts unknown effects based on the inferred cause, and induction tests the prediction and updates causal knowledge. A voluntary inquiry process is facilitated by epistemic emotions such as surprise, curiosity, interest, and confusion (Kashdan and Silvia, 2009; Vogl et al., 2020). Epistemic emotions drive exploratory behavior to obtain new information rather than pursue rewards. For example, psychologist Berlyne proposed epistemic curiosity, which promotes information-seeking behavior. He defined two types of epistemic curiosity: diversive and specific (Berlyne, 1966; Silvia, 2012). Diversive curiosity seeks novelty; thus, in this type of curiosity, surprise triggers abductive reasoning. On the other hand, specific curiosity drives induction, which seeks evidence of deductive reasoning to resolve confusion.

Emotions are generally mapped onto a dimensional space (Lang, 1995; Russell, 1980). The most commonly used dimensions are arousal and valence, termed the *core affect* (Russell, 2003). Arousal is the intensity of emotions, whereas valence is the dimension of the positive and negative poles. A recent functional magnetic resonance imaging (fMRI) study showed that arousal and valence are correlated with neural activity in the orbitofrontal cortex and amygdala, respectively (Wilson-Mendenhall et al., 2013). The emotional dimensions are not independent, and arousal affects valence. Berlyne's arousal potential theory suggests that an appropriate level of arousal potential induces a positive hedonic response, whereas extreme arousal induces a negative response (Berlyne, 1960). Thus, valence forms an inverse U-shaped function of the arousal potential, termed the *Wundt curve* (see Figure 4 in Berlyne, 1970). Berlyne suggests that epistemic curiosity approaches the optimal arousal potential, where the hedonic response (or positive valence) is maximized (Berlyne, 1960, 1966; Silvia, 2012).

Berlyne also illustrated a number of arousal potential factors, such as novelty, complexity, and uncertainty (Berlyne, 1960). Yanagisawa mathematically explains that the *free energy*, which is information on the brain's prediction error or Shannon's surprise (hereafter, surprise; Friston et al., 2006), represents the arousal potential because free energy is decomposed into information quantity terms representing perceived novelty, complexity, and uncertainty (Yanagisawa, 2021). This free-energy arousal model suggests that an appropriate level of free energy or surprise induces a positive emotional valence based on Berlyne's Wundt curve, which is supported by experimental evidence (Honda et al., 2022; Sasaki et al., 2024).

By contrast, the free energy principle (FEP; Friston et al., 2006), known as the unified brain theory (Friston, 2010), suggests that the brain must minimize its free energy during perception and action. Previous studies have proposed that decreasing and increasing free energy (or expected free energy) correspond to positive and negative valence, respectively (Clark et al., 2018; Hesp et al., 2021; Joffily and Coricelli, 2013; Seth and Friston, 2016; Wager et al., 2015; Yanagisawa et al., 2023), and that high and low free energies indicate uncertain and certain states, respectively. Reducing free energy resolves uncertainty and produces positive emotions.

The FEP argument that minimizing free energy corresponds to a positive valence seems to contradict the argument of arousal potential theory that an appropriate level of arousal potential [represented by free energy (Yanagisawa, 2021)] maximizes positive valence. To resolve this contradiction and integrate the FEP-based valence and arousal potential theories, we propose a novel valence framework based on the theory that a decrement in free energy and its expectation explains the valence of epistemic emotions. A decrease in free energy represents information gain and an epistemic value (Friston et al., 2017; Parr et al., 2022). The more information gain (epistemic value) one obtains or expects, the more positive the valence one experiences.

Based on this framework, we formulated emotion valence functions of arousal potential using reduction in free energy (i.e., information gains). Conventional Bayesian models (Section 3.1) that rely solely on Gaussian distributions predict infinite emotion valence as prediction error increases—an outcome that contradicts the known limits of human cognitive resources (Sweller, 2011; Taylor et al., 2022; Paas et al., 2003). The key novelty of this study lies in a simple modification: adding a uniform distribution to the likelihood function in a Gaussian generative model (Section 3.2). This modification models the influence of spontaneous neural firing activity (Raichle, 2006), which is thought to dominate in regions far from the mode of the likelihood function, where the Laplace approximation should not be applied. With this modeling, we show that the emotion valence function exhibits an inverted U-shape with respect to surprise, yielding a finite peak that aligns with the cognitive constraint of limited resources.

Furthermore, we analyzed the effects of prediction error and uncertainties on the peaks of the valence functions (Section 3.3). We associated epistemic emotions such as curiosity and interest with the free-energy-based valence model. Based on these analyses, we proposed an inquiry cycle model grounded in epistemic emotions derived from the free energy principle (Section 4.1).

The hypothesis of this study is as follows:

**Hypothesis:** By adding a uniform distribution to the likelihood function of a Gaussian generative model, the epistemic valence function exhibits an inverted U-shape with respect to surprise.

## 2 Materials and methods

### 2.1 Free energy formulations

FEP suggests that the brain must minimize its free energy through recognition, action, and learning (Friston et al., 2006). Assume an agent recognizes a hidden state *s* as a cause of a given observation *o*. We assume that the agent has a generative model *p*(*s, o*) as its knowledge about the probabilistic relationship between hidden states and observation and a recognition density *q*(*s*) of hidden states. The free energy is defined as a function of an observation representing the difference between a recognition density and a generative model averaged by the recognition density in terms of their energies (negative log probability).


(1) $$\begin{array}{c} F={\langle \ln q\left(s\right)-\ln p\left(s,o\right)\rangle }_{q\left(s\right)} \end{array}$$

The free energy represents the prediction error of recognition from the knowledge, i.e., the generative model. It refers to uncertainty and the prediction error of signals in a Bayesian brain theory (Knill and Pouget, 2004). The first and second terms on the right-hand side denote the negative-state entropy and internal energy, respectively. Thus, the definition corresponds to the Helmholtz free energy when the temperature is one.

With the definition of conditional probability, the generative model is factorized into true posterior and evidence: *p*(*s, o*) = *p*(*s*|*o*)*p*(*o*). With this factorization, the free energy is expanded to the summation of a Kullback–Leibler (KL) divergence and Shannon surprise (hereafter referred to as *surprise*).


(2) $$\begin{array}{c} F=D_{KL}\left[q\left(s\right)\|p\left(s|o\right)\right]-\ln p\left(o\right) \end{array}$$

The first-term KL divergence forms the true posterior to the recognition density, which represents a statistical difference between the two distributions: *D*_*KL*_[*q*(*s*)||*p*(*s*|*o*)] = 〈ln*q*(*s*)−ln*p*(*s*|*o*)〉_*q*(*s*)_. When the recognition approximates the true posterior to minimize free energy by the belief updating, the KL divergence becomes zero, and the free energy is approximated to the second term, i.e., surprise. Thus, the lower bound of free energy is surprise. Surprise is a negative log of the model evidence, *p*(*o*), and refers to the information content used to process given observations, representing cognitive load (Yanagisawa, 2021).

The generative model is decomposed to a state prior *p*(*s*) and a likelihood function *p*(*o*|*s*).


(3) $$\begin{array}{c} p\left(s,o\right)=p\left(s\right)p\left(o|s\right) \end{array}$$

With this decomposition, free energy is expanded to another two terms.


(4) $$\begin{array}{c} F=D_{KL}\left[q\left(s\right)\|p\left(s\right)\right]-{\langle \ln p\left(o|s\right)\rangle }_{q\left(s\right)} \end{array}$$

The first term is a KL divergence of state prior to recognition. This term represents the complexity of the generative model. The second term is the difference between likelihood and recognition. This term indicates negative model accuracy. Thus, minimizing the free energy signifies minimizing the complexity and maximizing the accuracy of the model.

### 2.2 Information gain in recognition

Assume that an initial recognition density before Bayesian belief-updating is approximated to the state prior *p*(*s*). The initial free energy *F*_0_ is a summation of KL divergence and surprise.


(5) $$\begin{array}{c} F_{o}={\langle \ln p\left(s\right)-\ln p\left(s,o\right)\rangle }_{p\left(s\right)}=D_{KL}\left[p\left(s\right)\|p\left(s|o\right)\right]-\ln p\left(o\right) \end{array}$$

The recognition density approximates the true posterior by minimizing the free energy. The KL divergence becomes zero, and the free energy reaches its lower bound *F*_*R*_, corresponding to surprise.


(6) $$\begin{array}{c} q\left(s\right):p\left(s\right)\to p\left(s|o\right),F_{R}=-\ln p\left(o\right) \end{array}$$

The decrease in free energy from before to after belief-updating in the recognition process is equivalent to the KL divergence from the true posterior to the initial recognition, *KLD*. Herein, *KLD* denotes the information gain from recognizing the causal state of a given observation.


(7) $$\begin{array}{c} \Delta F_{R}=F_{0}-F_{R}=KLD=D_{KL}\left[p\left(s\right)\|p\left(s|o\right)\right] \end{array}$$

A greater KLD indicates that the recognition of an observation under a policy provides greater information gain. Thus, KLD represents the epistemic value of recognizing an observation. This suggests that an agent prefers to recognize observations with a greater KLD. Therefore, we infer that KLD increases positive valence by increasing information gain (epistemic value) in recognition.

### 2.3 Information gain expected from Bayesian updating prior belief: Bayesian surprise

The free energy minimized by a recognition, *F*_*R*_, approximates surprise. The minimized free energy equals the sum of complexity and inverse accuracy with a recognition approximated to the true posterior, *q*(*s*) ≈ *p*(*s*|*o*).


(8) $$\begin{array}{c} F_{R}=-\ln p\left(o\right)=BS+U \end{array}$$

(9) $$\begin{array}{c} BS=D_{KL}\left[p\left(s|o\right)\|p\left(s\right)\right] \end{array}$$

(10) $$\begin{array}{c} U=-{\langle \ln p\left(o|s\right)\rangle }_{p\left(s|o\right)} \end{array}$$

The complexity and inverse accuracy terms represent the Bayesian surprise *BS* and perceived uncertainty *U*, respectively, and their summation (surprise) denotes the arousal potential (Yanagisawa, 2021). The Bayesian surprise, *BS*, is a KL divergence from posterior to prior, i.e., the deviation of recognition from prior expectation of states. The difference between surprise and BS is that the former is information about observation, whereas BS is information gain about states. BS represents the novelty of the recognized observation and is correlated with the human surprise response to novel stimuli (Yanagisawa et al., 2019). This may be because states are perceivable, but observations are not. The surprise response decreases with repeated exposure to the same novel stimuli. Such habituation is formulated as a decrease in *BS* in the Bayesian update of the prior (Ueda et al., 2021).

When handling multiple, sequential, observations *o*_1_, *o*_2_, which are assumed to be independent and identically distributed (i.i.d.) and generated from the same hidden state, *s*, the computation of the posterior distribution *p*(*s* |*o*_1_, *o*_2_) can be approached in two ways. The first approach is to compute the posterior in a single stem as: *p*(*s* |*o*_1_, *o*_2_) = *p*(*s*) *p*(*o*_1_ | *s*) *p*(*o*_2_ | *s*)/*p*(*o*_1_, *o*_2_). Alternatively, this process can be interpreted as a two-step update. In the first step, the posterior based on the initial observation is computed: *p*(*s* |*o*_1_) = *p*(*s*) *p*(*o*_1_| *s*)/*p*(*o*_1_). Subsequently, the posterior is updated based on the second observation: *p*(*s*|*o*_1_, *o*_2_) = *p*(*s* |*o*_1_) *p*(*o*_2_ | *s*)/*p*(*o*_2_ | *o*_1_). In this formulation, the posterior distribution obtained from the first observation effectively serves as the prior for the second update. In this study, we adopt the latter, sequential interpretation. This perspective implies that following the first update—prior to observing *o*_2_–the initial prior *p*(*s*) is effectively replaced by the posterior *p*(*s* ∣ *o*_1_). Furthermore, the KL divergence associated with this update becomes zero, since the approximate posterior *q*(*s*) is identical to the true posterior after the first update: (i.e., *BS* = *D*_*KL*_[*q*(*s*)||*p*(*s*|*o*_1_)] = 0, where *q*(*s*) = *p*(*s*|*o*_1_)). Consequently, the free energy decreases to the inverse accuracy term. We refer to this term as *uncertainty* because it refers to the perceived uncertainty (Yanagisawa, 2021). Thus, the lower bound of free energy after the prior updating is the uncertainty, *F*_*L*_ ≤ *U*, whereas the upper bound of the free energy decrease is the Bayesian surprise, *BS*.


(11) $$\begin{array}{c} \Delta F_{L}=F_{R}-F_{L}\leq BS \end{array}$$

Herein, *BS* is equivalent to the maximum information gain expected from the prior update based on i.i.d observations. A greater *BS* denotes a greater information gain expected from the update. Thus, BS represents the expected epistemic value given by the model (prior) update or learning. This suggests that an agent prefers novel observations with a greater *BS*, which is expected to provide a chance to learn new information (update its own generative model). Therefore, we infer that BS increases emotional valence in anticipation of information gain from updating prior beliefs.

### 2.4 Linking free energy reduction, information gain, and arousal potential

The free energy given an observation *o* decreases by *KLD* as the first information gain when one succeeds in recognizing the state as a cause of the observation. The minimized free energy approximates surprise. The surprise is a summation of *BS* and *U*. When one's prior is updated to the posterior, the free energy is decreased by *BS*, which is the expected second information gain.

The upper bound of the total free energy reduction (or information gain) from recognizing and updating state beliefs, given an observation, is a summation of the two KL divergences, i.e., information gain.


(12) $$\begin{array}{c} \Delta F_{R}+\Delta F_{L}\leq \;KLD+BS=D_{KL}\left[p\left(s\right)\|p\left(s|o\right)\right]\\ +D_{KL}\left[p\left(s|o\right)\|p\left(s\right)\right]\equiv IG \end{array}$$

**Figure 1:**
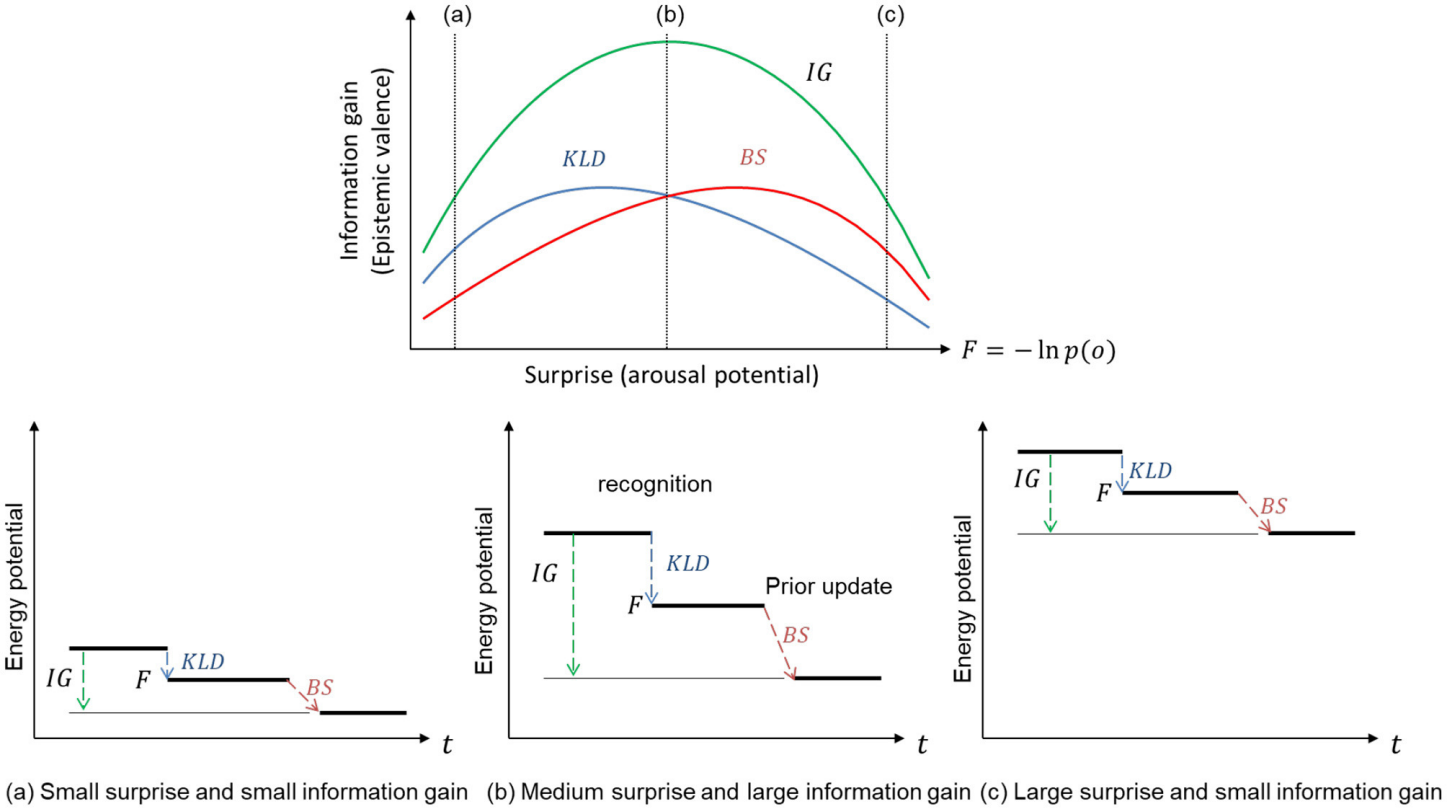
Relationship between information gain as epistemic value and surprise as arousal potential. An appropriate level of surprise provides a large information gain that decreases free energy potential.

We consider that the total information gain represents the epistemic values that explain the emotional valence of the arousal potential. Figure 1 shows the link between the two types of KL divergences (KLD and BS), as well as the total information gains (*IG*) and inverse-U-shaped function of arousal potential (*F*).

The two types of KL divergence denote the difference between the prior and posterior. When the posterior given observation is the same as the prior, the KL divergences are zero, and the observation provides minimum free energy and minimum surprise (or maximum evidence). Hence, an observation that provides minimal free energy does not provide any KL divergence or information gain. When the prediction error and surprise (minimized free energy) are small, both KLD and BS are small, and thus, the information gain or epistemic value-based valence is small [Figure 1 (a)]. To provide epistemic value with an emotional valence, given information gain, a certain level of surprise representing arousal potential (Yanagisawa, 2021) is required by observing unexpected outcomes that give certain KL divergences [Figure 1 (b)]. However, if the likelihood of an observation is far from the prior distribution, where the likelihood does not provide any information, the posterior is not updated from the prior. In this case, the KL divergences are small, and the observation provides little information [Figure 1 (c)]. Therefore, we consider that an appropriate level of surprise maximizes the KL divergences (information gains) and that an appropriate level represents the optimal arousal potential that maximizes the positive valence for its epistemic value.

KL divergence is an asymmetric operation. Hence, although both KL divergences, *KLD* and *BS*, denote differences between the prior and posterior, they are different from each other. This suggests that the two KL divergences as functions of surprise are different. KLD signifies information gain due to recognition, whereas BS signifies maximum information gain expected from updating prior beliefs based on i.i.d. observations. This suggests that maximizing KLD and BS are different strategies for approaching the optimal arousal level that maximizes the total epistemic value with a positive valence.

### 2.5 Analytical methodology

We modeled the two information gains, KLD and BS, as functions of surprise using a Gaussian-like generative model with a flat likelihood of uniform noise and demonstrated that the two functions, KLD and BS, form an inverse-U shape and have different peaks. Using the function model, we analyzed the effect of Gaussian parameters, the difference between the prior mean and likelihood peak as *prediction error* (Yanagisawa, 2016), variance of prior as *prediction uncertainty*, and variance of likelihood as *observation uncertainty* on the peaks of the information gain functions. From the analysis, we elucidated the conditions for optimal prediction errors and uncertainties of prediction and observation to maximize the information gains in an ideal inquiry process.

## 3 Results

### 3.1 Gaussian model of information gains

In this section, we derive the standard expressions of information gain without applying the key assumption of this study, adding a uniform distribution to the likelihood function. The results with the uniform distribution added are presented in Section 3.2.

In our analysis, we adopted the Gaussian Bayesian model. The Gaussian Bayesian model has been used in past research studies to analyze the characteristics of free energy and Bayesian surprise (Buckley et al., 2017; Yanagisawa et al., 2023; Yanagisawa, 2021). The Laplace approximation suggests that a Gaussian distribution is applied around the mode of unknown distributions. Furthermore, the Gaussian form allows independent manipulation of the mean and variance of the distribution, making it well-suited for the purpose of this analysis, which aims to examine how the mean and variance affect the KL divergence.

The distance between the prior mean and likelihood peak, δ, represents *prediction error*; the variance of prior $\sigma_{p}^{2}$, represents *prior uncertainty*; and the variance of likelihood $\sigma_{l}^{2}$, represents *observation uncertainty*. With a Gaussian likelihood function $p\left(o|s\right)=N\left(o,\sigma_{l}^{2}\right)$ and a Gaussian prior distribution $p\left(s\right)=N\left(\eta ,\sigma_{p}^{2}\right)\equiv N_{pri}$, the posterior distribution is *p*(*s*|*o*) = *N*(η_*post*_, *s*_*post*_) ≡ *N*_*post*_, where $s_{post}=\frac{\sigma_{p}^{2}\sigma_{l}^{2}}{\sigma_{p}^{2}+\sigma_{l}^{2}}$ and $\eta_{post}=\frac{\sigma_{p}^{2}o+\sigma_{l}^{2}\eta }{\sigma_{p}^{2}+\sigma_{l}^{2}}$ [see Equation 5 in Yanagisawa et al. (2019)].

The evidence *p*(*o*) is a marginal likelihood:


(13) $$\begin{array}{c} p\left(o\right)=\int_{-\infty }^{\infty }p\left(o|s\right)p\left(s\right)ds=\exp\left[-\frac{\delta^{2}}{2\left(\sigma_{p}^{2}+\sigma_{l}^{2}\right)}\right]\equiv e\left(\delta \right), \end{array}$$

where δ = η−*o* is a prediction error. The evidence *e*(δ) is an inverse exponential function of the square of the prediction error. The evidence exponentially decreases as the prediction error increases. Surprise, the lower bound of free energy, is a negative log function of evidence, i.e., −log*p*(*o*) = −log*e*(δ). Thus, the surprise is a quadratic function of a prediction error.

Using this Gaussian model, we derive the information gains, KLD and BS, as quadratic functions of the prediction error δ with the coefficients of variance:


(14) $$\begin{array}{c} KLD_{N}=D_{KL}\left(p\left(s\right)\|p\left(s|o\right)\right)=A_{KLD}\delta^{2}+B_{KLD}, \end{array}$$

where the coefficients are $A_{KLD}=\frac{\sigma_{p}^{2}}{2\sigma_{l}^{2}\left(\sigma_{p}^{2}+\sigma_{l}^{2}\right)}$ and $B_{KLD}=-\ln\frac{{\sigma_{p}^{2}+\sigma }_{l}^{2}}{\sigma_{l}^{2}}+\frac{\sigma_{p}^{2}}{\sigma_{l}^{2}}$; and


(15) $$\begin{array}{c} BS_{N}=D_{KL}\left(p\left(s|o\right)\|p\left(s\right)\right)=A_{BS}\delta^{2}+B_{BS}, \end{array}$$

where the coefficients are $A_{BS}=\frac{\sigma_{p}^{2}}{2{\left({\sigma_{p}^{2}+\sigma }_{l}^{2}\right)}^{2}}$ and $B_{BS}=\ln\frac{{\sigma_{p}^{2}+\sigma }_{l}^{2}}{\sigma_{l}^{2}}-\frac{\sigma_{p}^{2}}{{\sigma_{p}^{2}+\;\sigma }_{l}^{2}}$.

Since both *A*_*KLD*_ and *A*_*BS*_ are positive, prediction error always increases both KLD and BS.

### 3.2 Convexity of information gain function by considering uniform noise

This section presents the analytical results based on the hypothesis of this study, in which a uniform distribution is added to the Gaussian likelihood function.

The results in Section 3.1 suggest that, under the assumption of a Gaussian likelihood function, information gain continues to increase indefinitely as prediction error grows. However, this prediction appears unrealistic when we consider the widely accepted view that human cognitive resources are limited (Sweller, 2011; Taylor et al., 2022; Paas et al., 2003).

This unrealistic outcome stems from applying the Laplace approximation even in regions with large prediction errors, that is, areas far from the mode of the likelihood function.

The Laplace approximation approximates the shape of a probability distribution near its mode (the most frequent value) using a Gaussian distribution. Researchers should, therefore, restrict its use to analyses around the mode. In fact, many active inference studies (Baioumy et al., 2021; Lanillos et al., 2021; Priorelli and Stoianov, 2023) apply this approximation based on the implicit assumption that neural activity concentrates near the mode.

In contrast, this study focuses on information gain in conditions with large prediction errors—that is, regions far from the mode—where the assumptions behind the Laplace approximation may no longer hold.

The model used in Section 3.1, which assumes a Gaussian likelihood function, has limitations when viewed considering neuroscientific theories and empirical findings. According to the rate-coding hypothesis, the likelihood function is encoded by the distribution of neuronal firing rates. As the input moves away from the mode, the value of the likelihood decreases exponentially, approaching zero. If we align this behavior with the rate-coding hypothesis, we must conclude that in regions far from the mode, neurons almost completely stop firing—their firing rates approach zero. However, empirical research in neuroscience (Raichle, 2006; Destexhe et al., 2003) shows that neurons fire spontaneously at low frequencies, even without external stimuli. In other words, spontaneous spiking activity continues in the brain even when no external input is present. Therefore, a model that uses only a Gaussian likelihood function fails to reflect this spontaneous neural activity.

This observation leads us to believe that, in regions far from the mode, spontaneous activity—independent of external stimuli—plays a more dominant role than stimulus-driven neural responses, which tend to vanish. In this section, we aim to describe information gain under large prediction errors more realistically. To do so, we must better model the influence of neural activity in regions where the Laplace approximation breaks down. Specifically, following the approach of Jones (2016), we added an independent uniform distribution with a very small constant probability ε to the likelihood function to capture the effect of spontaneous neural activity.


(16) $$\begin{array}{c} p_{\varepsilon }\left(o|s\right)=\alpha \left(p\left(o|s\right)+\varepsilon \right),\; \end{array}$$

where α = 1/(1 + ε) is a coefficient for standardization.

**Figure 2:**
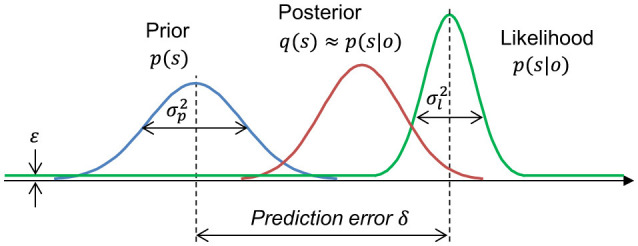
Gaussian Bayesian model with uniform likelihood. $\sigma_{p}^{2}$: prior variance, $\sigma_{l}^{2}$: Gaussian likelihood variance, δ: prediction error, and ε: probability of uniform likelihood.

This uniform likelihood addition flattens the tail of the Gaussian likelihood function, as shown in Figure 2. The effect of the Gaussian tail becomes negligible as the prediction error increases. Therefore, we infer that adding uniform likelihood is the simplest modeling method to represent the likelihood of spontaneous neural activity and to ignore the effect of the Gaussian likelihood tail.

The evidence with the likelihood function is the Gaussian evidence and the constant probability.


(17) $$\begin{array}{c} p_{\varepsilon }\left(o\right)=\int_{-\infty }^{\infty }p_{\varepsilon }\left(o|s\right)p\left(s\right)ds=\alpha \left(e\left(\delta \right)+\varepsilon \right)\; \end{array}$$

Notably, surprise increases monotonically with respect to the prediction error. We find that the posterior distributions with the likelihood function form a weighted linear model of the Gaussian posterior and prior.


(18) $$\begin{array}{c} p_{\varepsilon }\left(s|o\right)=\frac{p\left(s\right)p_{\varepsilon }\left(o|s\right)}{p_{\varepsilon }\left(o\right)}=\frac{\alpha \left(e\left(\delta \right)N_{post}+\varepsilon N_{pri}\right)}{\alpha \left(e\left(\delta \right)+\varepsilon \right)}\\ =w_{post}N_{post}+w_{pri}N_{pri}, \end{array}$$

where $w_{post}=\frac{e\left(\delta \right)}{e\left(\delta \right)+\varepsilon }$ and $w_{pri}=\frac{\varepsilon }{e\left(\delta \right)+\varepsilon }$ are the standardized linear weights. When the prediction error is small, the term ε*N*_*pri*_ is negligible because ε is very small compared to *e*(δ). In this case, the posterior is approximated to the Gaussian posterior, *p*_ε_(*s*|*o*) ≈ *N*_*post*_. Thus, the prediction error increases both information gains, KLD and BS. By contrast, when the prediction error increases toward infinity, the evidence converges to zero, $\underset{\delta \to \infty }{\text{lim}}e\left(\delta \right)=0$ because the evidence is the inverse exponential function of the prediction error. In this case, the Gaussian posterior is negligible, and thus, the posterior is approximated to the Gaussian prior, $\underset{\delta \to \infty }{\text{lim}}p_{\varepsilon }\left(s|o\right)=N_{pri}$. When the posterior is equal to the prior, both information gains, KLD and BS, are zero. Thus, in the case of a large prediction error, where *e*(δ) is very small compared to ε, and ε*N*_*pri*_ is dominant in the posterior, the information gains decrease to zero as prediction error increases toward infinity. We use ε = 10^−3^ for the following analysis.

**Figure 3:**
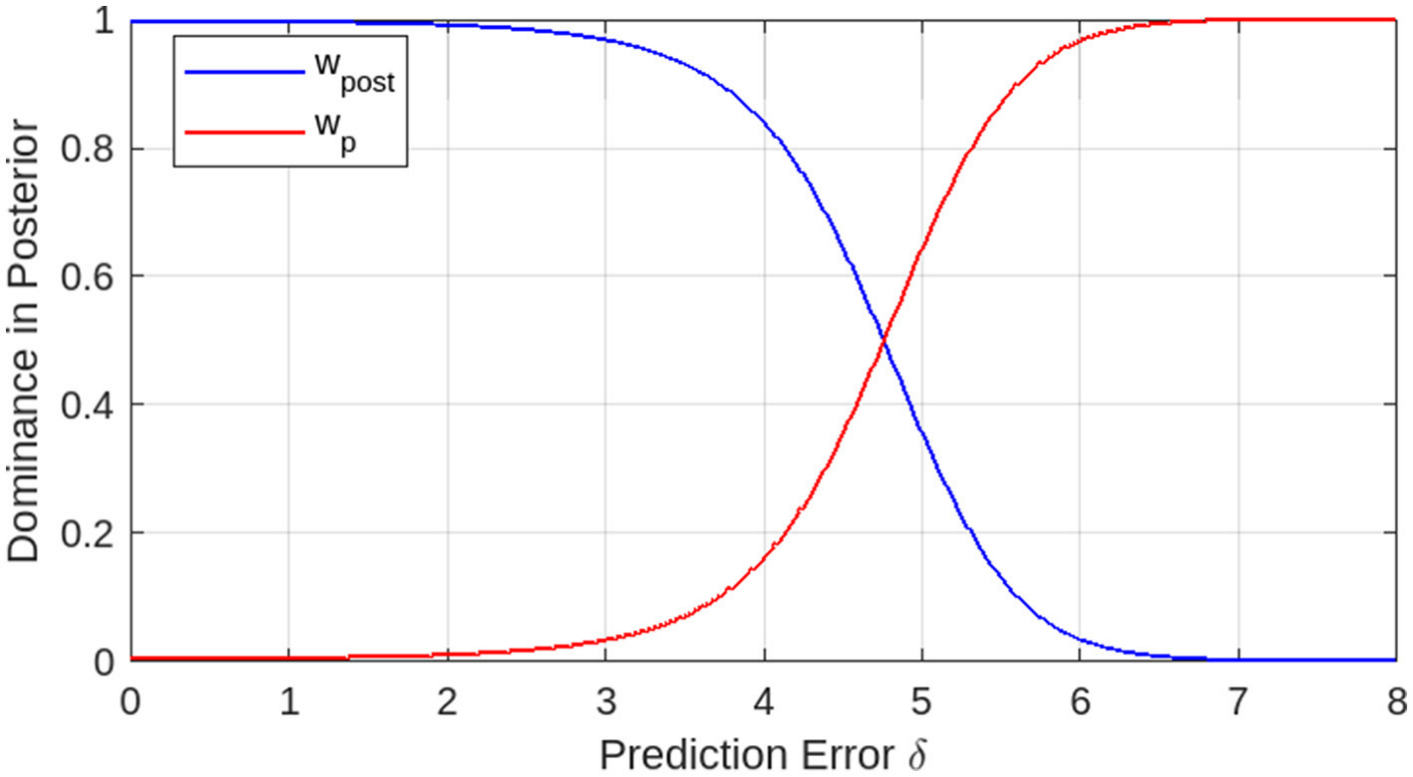
Dominance of Gaussian posterior and prior in posterior distribution as functions of prediction error. The dominances switch over at a certain prediction error level (Variances: $\sigma_{p}^{2}=10.0,\;\sigma_{l}^{2}=$1.0.).

The standardized linear weights *w*_*post*_ and *w*_*pri*_ represent the dominance of the Gaussian posterior and prior, respectively, in the mixed posterior distribution. Figure 3 shows the dominances as functions of prediction error δ. When the prediction error is zero or small, the Gaussian posterior is dominant. For a certain prediction error, the Gaussian prior becomes dominant as the prediction error increases. In the switching over area of prediction errors, the Gaussian posterior and prior are mixed with certain weights, *w*_*post*_ and *w*_*pri*_.

Using the posterior function, we derive KLD and BS (see Appendix). Since the computed results include integrals that cannot be solved analytically, we used a computational approach for further analysis.

**Figure 4:**
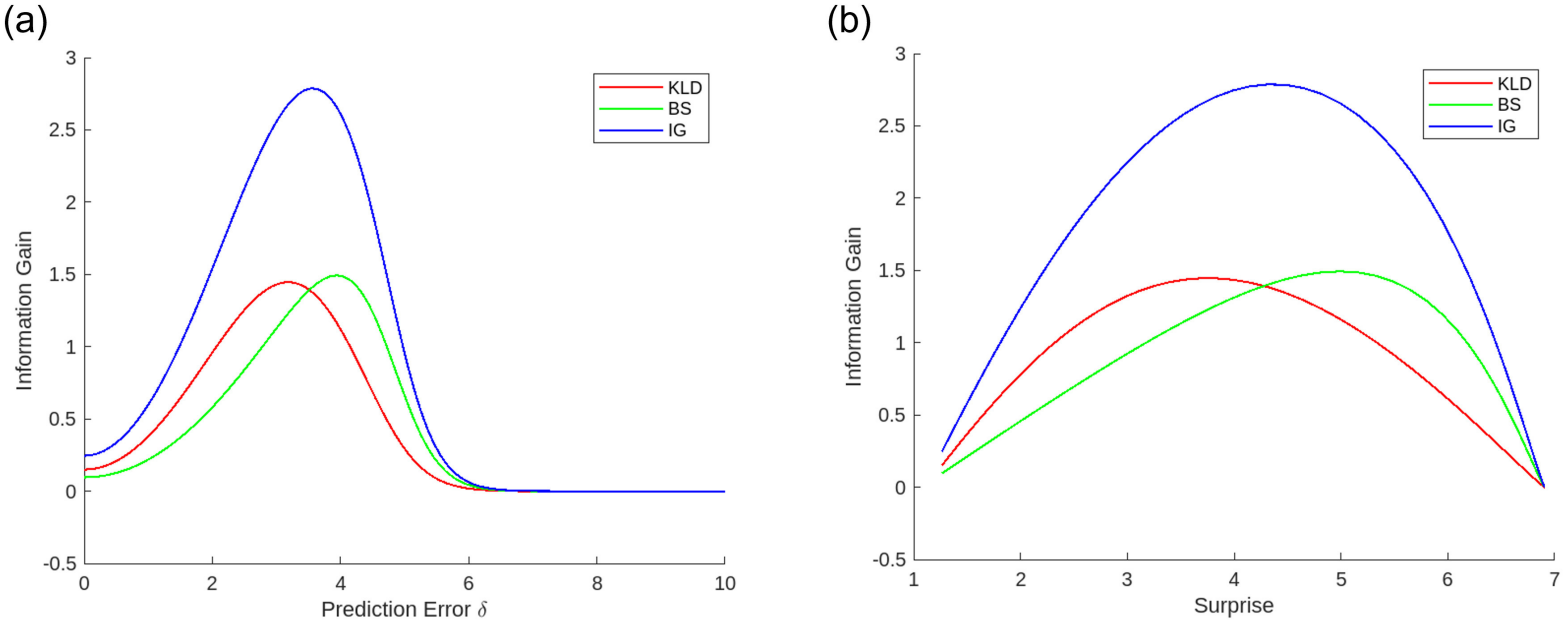
Example of information gain functions of **(a)** prediction error and **(b)** surprise using Gaussian model with uniform noise. KLD and BS represent free energy reduction in recognition and prior updating (learning), respectively. Total information gain *IG* is a summation of KLD and BS (Variances: $\sigma_{p}^{2}=10.0,\;\sigma_{l}^{2}=$1.0.).

Figure 4a shows the information gains and their total value, *IG* = *KLD* + *BS*, as functions of the prediction errors. All information gains are upward-convex functions of the prediction errors. This convexity is general because when the prediction error is small, the Gaussian posterior is dominant in the posterior, and information gains increase as the prediction error increases; whereas when the prediction error is larger than a certain level, the prior becomes dominant, and the information gains decrease to zero as the prediction error increases.

The surprise obtained by taking the negative logarithm of Equation 17 was found to increase monotonically with prediction errors. Thus, the information gains are also upward-convex functions of surprise, and the total information gain *IG* that induces positive emotions by reducing free energy is an upward-convex function of surprise (and prediction error). We infer that the upward-convex function of the total information gain represents the arousal potential function (i.e., the Wundt curve). Figure 4b shows an example of information gain as a function of surprise.

Information gain functions are upward-convex and have a peak. We define the prediction errors that maximize information gains *KLD*, *BS*, and *IG* as *optimal prediction errors* δ_*KLD*_, δ_*BS*_ and δ_*IG*_, respectively. Similarly, we define the surprises that maximize information gains *KLD*, *BS*, and *IG* as *optimal surprises* *S*_*KLD*_, *S*_*BS*_, and *S*_*IG*_, respectively. We use the term “optimal” because it represents the optimal arousal level that maximizes information gain (epistemic value) that evokes emotional valence. When the prediction errors are greater than δ_*KLD*_ and smaller than δ_*BS*_, *KLD* and *BS* have a negative relationship, where *KLD* decreases as *BS* increases, and vice versa. The prediction error that maximizes the total information gain δ_*IG*_ always falls into this area. Alternate maximizations of *KLD* and *BS* by decreasing and increasing the prediction error and surprise in this area iteratively reach the optimal surprise *S*_*IG*_. This alternation generates fluctuations of surprise. The magnitude of fluctuation is determined by the difference between KLD and BS in the optimal prediction error *D*_δ_ = δ_*BS*_ − δ_*KLD*_ and surprise *D*_*S*_ = *S*_*BS*_ − *S*_*KLD*_. In the next section, we analyze the effects of uncertainties on the optimal prediction errors and surprise, together with their differences.

### 3.3 Effects of uncertainties on information gains

The optimal prediction error and surprise change depending on uncertainties. We found the optimal prediction error and optimal surprise for all combinations of likelihood variances $\sigma_{l}^{2}$ [1.0, 50] and prior variance $\sigma_{p}^{2}$ [1.0, 50] in steps of 0.1 using the MATLAB fminbnd.m function, which is based on golden section search and parabolic interpolation.

**Figure 5:**
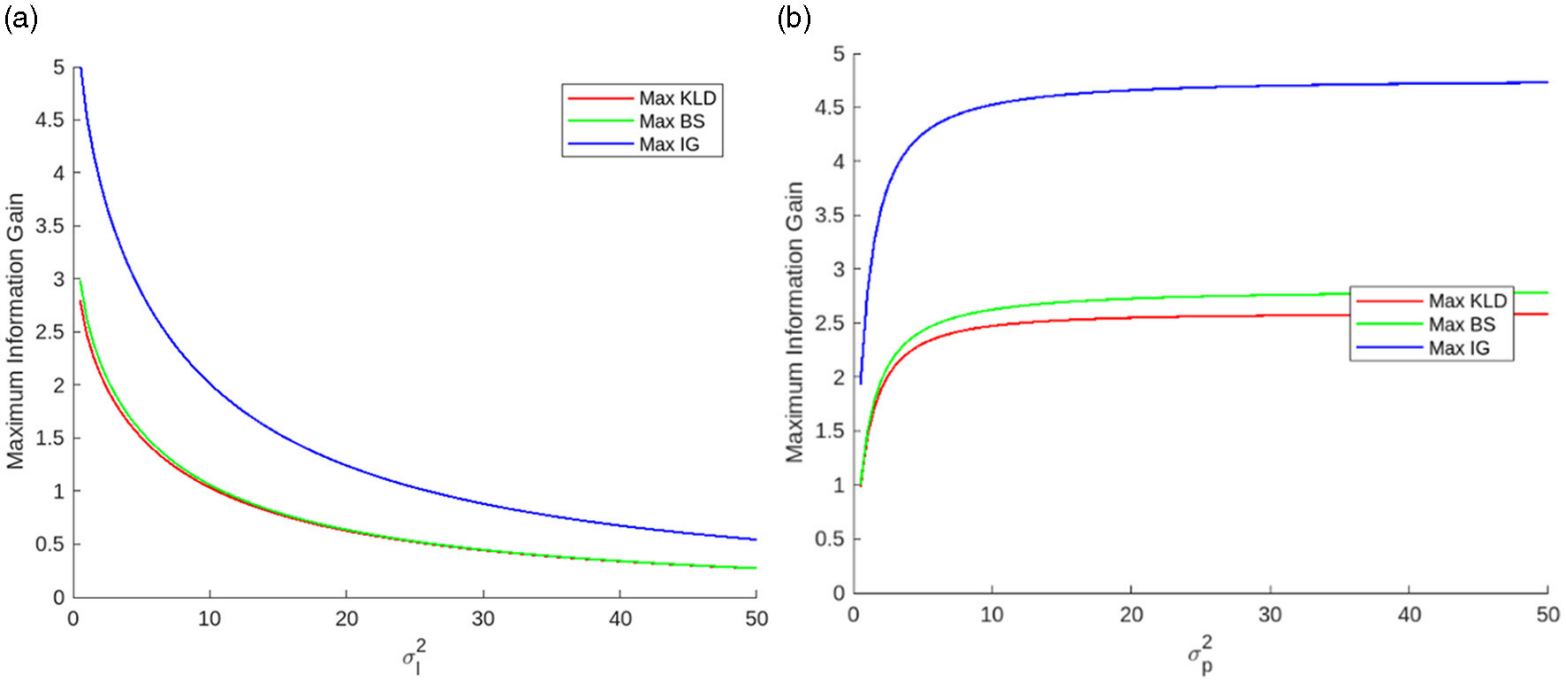
Maximum information gains as functions of **(a)** likelihood variance when $\sigma_{p}^{2}=10$ and **(b)** prior variance when $\sigma_{l}^{2}=1.0$.

Figure 5 shows examples of the maximum information gains as a function of $\sigma_{l}^{2}$ and $\sigma_{p}^{2}$. The maximum information gains increase exponentially as $\sigma_{l}^{2}$ decreases. Thus, the sensitivity of $\sigma_{l}^{2}$ to the maximum information gains increases as $\sigma_{l}^{2}$ decreases. By contrast, the sensitivity of $\sigma_{p}^{2}$ to information gain is significant when $\sigma_{p}^{2}$ is small (e.g., from 1.0 to 10.0 in this example).

**Figure 6:**
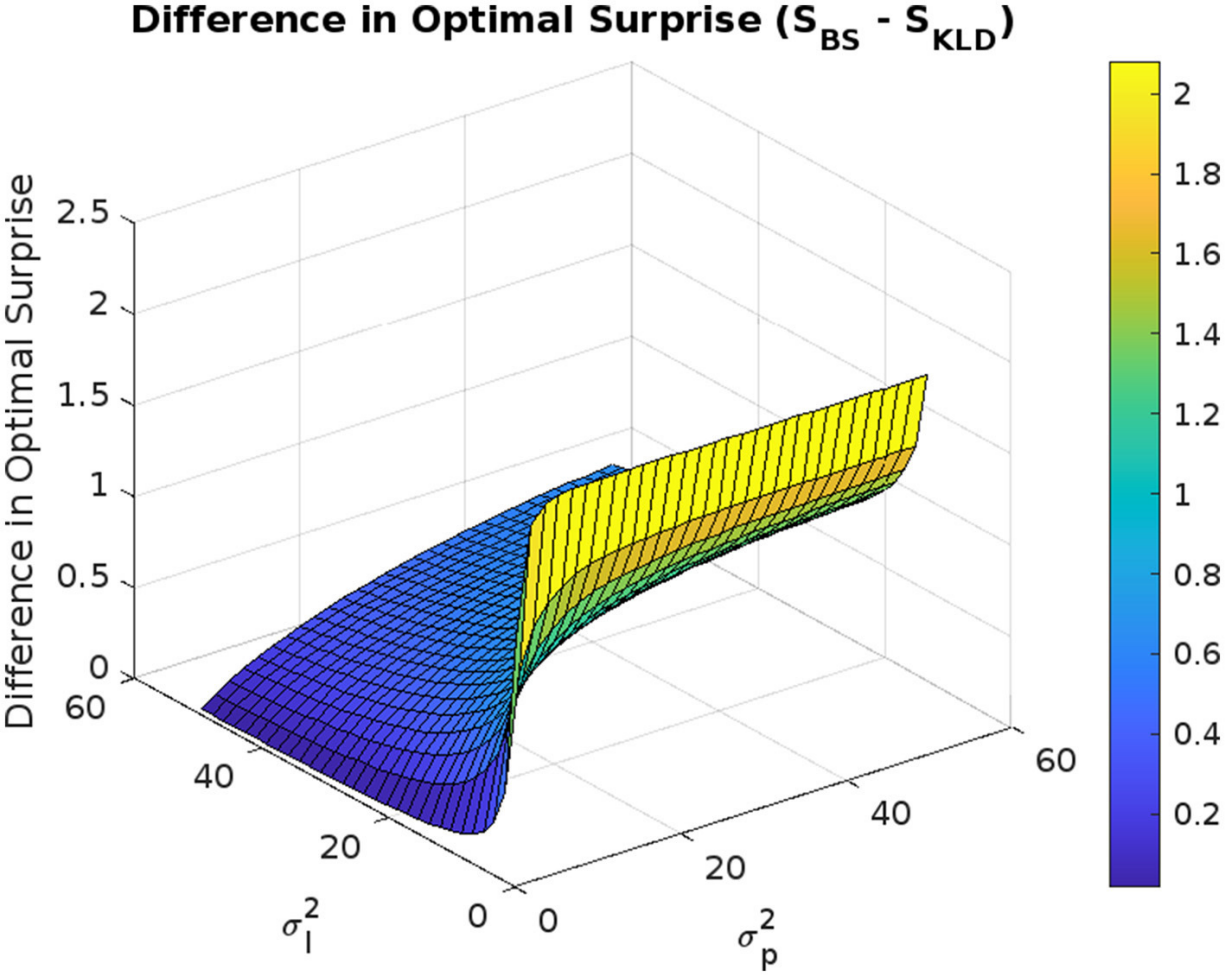
Difference in optimal surprise *D*_*S*_ as a function of observation and prediction uncertainties.

We omit the figure for the analysis in which both $\sigma_{l}^{2}$ and $\sigma_{p}^{2}$ were varied simultaneously, but we describe the outline of the results below. While $\sigma_{p}^{2}$ approaches zero, the sensitivity of $\sigma_{l}^{2}$ to the maximum information gains is low. The sensitivity of $\sigma_{l}^{2}$ increases as $\sigma_{p}^{2}$ increases. The peak of the maximum information gain is observed when $\sigma_{l}^{2}$ is small, and $\sigma_{p}^{2}$ is large. The maximum information gain of a large $\sigma_{l}^{2}$ and large $\sigma_{p}^{2}$ is greater than that of a small $\sigma_{l}^{2}$ and small $\sigma_{p}^{2}$. These trends are similar to those shown in Figure 6 (the difference in the optimal surprise *D*_*S*_).

Figure 6 shows the difference in the optimal surprise *D*_*S*_ = *S*_*BS*_ − *S*_*KLD*_. The difference *D*_*S*_ is always positive, and thus, *S*_*BS*_ > *S*_*KLD*_. *D*_*S*_ increases as $\sigma_{l}^{2}$ decreases and $\sigma_{p}^{2}$ increases. Thus, the larger the $\sigma_{p}^{2}$, and the smaller the $\sigma_{p}^{2}$, the larger *D*_*S*_. $\sigma_{l}^{2}$ has the greatest sensitivity to increase *D*_*S*_ when $\sigma_{p}^{2}$ is large. *D*_*S*_ is larger when both $\sigma_{l}^{2}$ and $\sigma_{p}^{2}$ are small than when both $\sigma_{l}^{2}$ and $\sigma_{p}^{2}$ are large.

**Figure 7:**
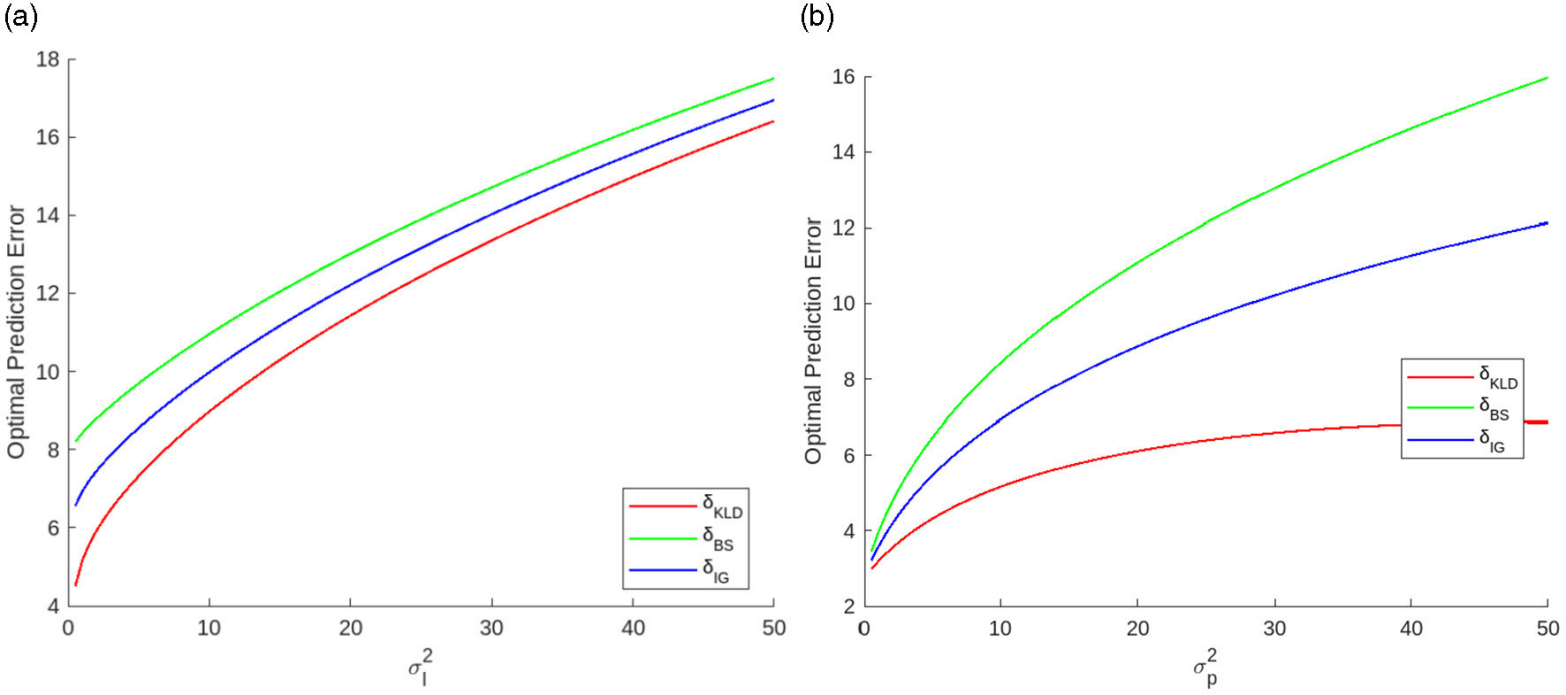
Optimal prediction errors as functions of uncertainties, **(a)** likelihood variance when $\sigma_{p}^{2}=$10.0 and **(b)** prediction variance when $\sigma_{l}^{2}=$1.0.

Figure 7 shows the optimal prediction errors as a function of each variance. All functions are monotonically increasing convex. δ_*KLD*_ is more sensitive to $\sigma_{l}^{2}$ than δ_*BS*_. Thus, the difference between δ_*KLD*_ and δ_*BS*_ decreases as $\sigma_{l}^{2}$ increases. By contrast, δ_*KLD*_ is less sensitive to $\sigma_{p}^{2}$ than δ_*BS*_. Thus, the difference between δ_*KLD*_ and δ_*BS*_ increases as $\sigma_{p}^{2}$ increases.

**Figure 8:**
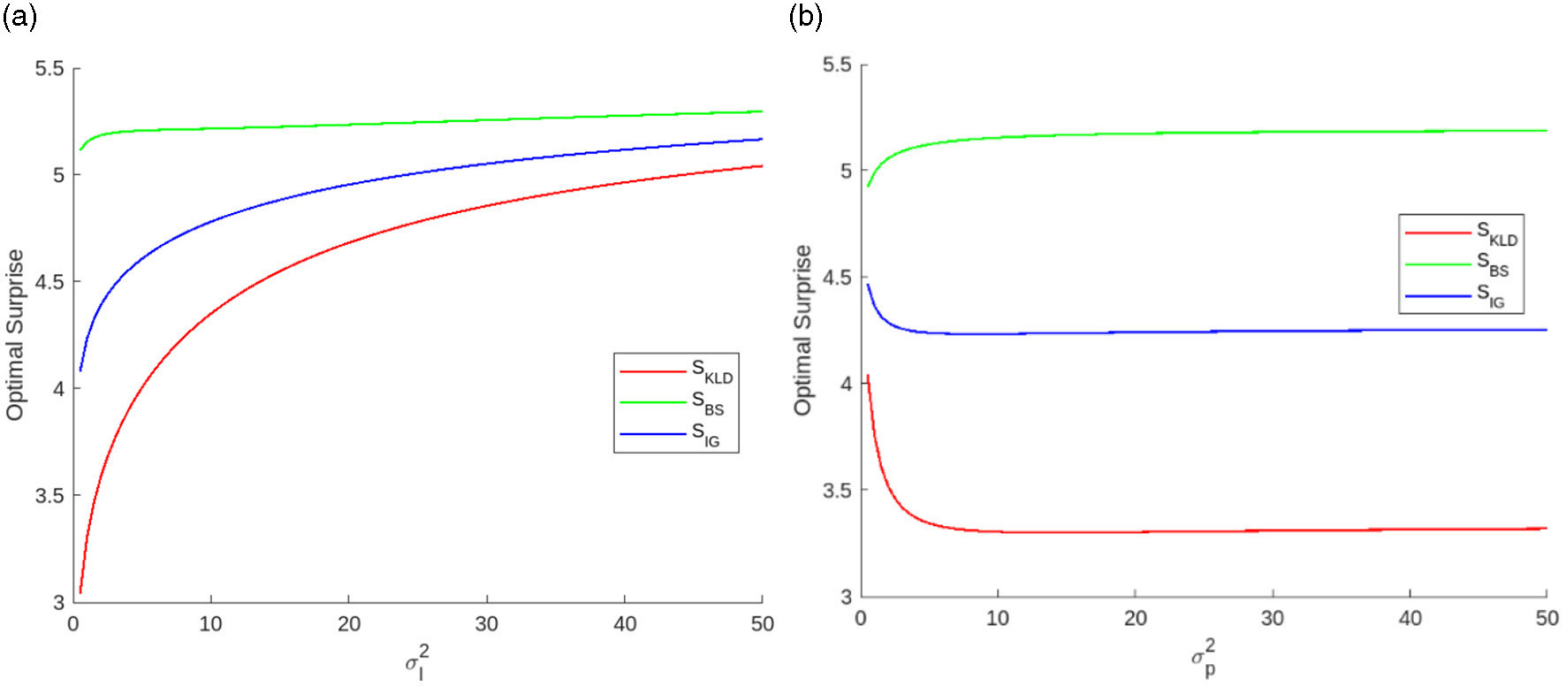
Optimal surprises as functions of **(a)** likelihood variance when $\sigma_{p}^{2}=10.0$ and **(b)** prediction variance when $\sigma_{l}^{2}=1.0$.

Figure 8 shows examples of optimal surprises as functions of each variance. $\sigma_{l}^{2}$ monotonically increases all optimal surprises. However, the effects of $\sigma_{p}^{2}$ are different. $\sigma_{p}^{2}$ decreases *S*_*KLD*_ and increases *S*_*BS*_.

## 4 Discussions

### 4.1 Arousal potential functions and curiosities

**Figure 9:**
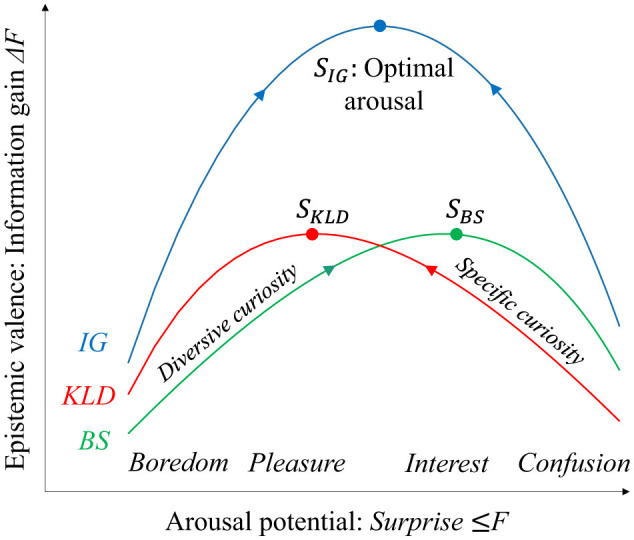
Schematic of arousal potential functions and related emotions. The valence of epistemic emotions represented by information gains forms the upward function of arousal potential represented by free energy or surprise. Diversive and specific curiosity drive to maximize KLD and BS, respectively. These alternate maximizations achieve optimal arousal levels with fluctuation of surprise. Emotions such as boredom, pleasure, interest, and confusion are induced by free energy and its fluctuations (see main text for detailed discussion).

The results of the analysis using a Gaussian generative model with an additional uniform likelihood suggest that the two information gains, KLD and BS, form upward-convex functions of surprise and prediction errors (i.e., the distance between the prior mean and likelihood peak). The prediction error monotonically increases surprise. Figure 9 shows a schematic of the information gain functions that conceptualize the analytical results, as shown in Figure 4b and the related emotions. Surprise, −ln*p*(*o*), corresponds to free energy minimized in recognition. A previous study argued that surprise represents arousal potential because minimized free energy consists of the summation of information content provided by novelty and perceived complexity, which are collative variables and dominant factors of arousal potential (Yanagisawa, 2021).

Berlyne suggested that an appropriate level of arousal potential induces a positive hedonic response, termed the optimal arousal level (Berlyne, 1960). Extreme arousal level caused by novel and complex stimuli may cause confusion. By contrast, a low arousal level with familiar and simple stimuli results in boredom. Thus, emotional valence shapes the upward-convex function of the arousal potential, termed the Wundt curve.

Berlyne also suggested that two epistemic curiosities, diversive and specific, drive the approach to the optimal arousal level (Berlyne, 1966). Diversive curiosity drives the pursuit of novelty, whereas specific curiosity drives the search for evidence of one's model predictions. Consequently, diversive curiosity increases the arousal potential to climb the Wundt curve on the left, from a low level of arousal (boredom). By contrast, specific curiosity motivates a decrease in the arousal potential to climb the Wundt curve on the right side from a high arousal level (confusion). The alternation between the two curiosity-driven activities approaches the optimal arousal level.

*KLD* is a free energy reduction in recognition of a state *s* given an observation *o* that increases model evidence, *p*(*o*) = 〈*p*(*o*|*s*)〉_*q*(*s*)_, where recognition *q*(*s*) is updated from a prior *p*(*s*) to true posterior *p*(*s*|*o*). *BS* is the expected information gain given by novel stimuli that correspond to human surprise response to novelty (Itti and Baldi, 2009; Sekoguchi et al., 2019; Ueda et al., 2021; Yanagisawa et al., 2019). Therefore, we consider that specific curiosity drives an increase in KLD, whereas diversive curiosity drives an increase in BS.

### 4.2 Inquiry process and epistemic emotions

The analytical result shown in Figure 6 demonstrates that the optimal surprise for BS is always greater than that of KLD, i.e., *S*_*BS*_ > *S*_*KLD*_. This result suggests that maximizing information gain through novelty seeking (driven by diversive curiosity) requires a higher level of surprise compared to maximizing information gain through evidence seeking (driven by specific curiosity).

When surprise is less than *S*_*KLD*_, both KLD and BS monotonically increase as surprise increases. By contrast, when surprise is greater than *S*_*BS*_, both KLD and BS monotonically decrease as surprise increases. Thus, the two curiosities respectively increase and decrease prediction errors in the former and latter areas of surprise, respectively. However, when surprise is greater than *S*_*KLD*_ and less than *S*_*BS*_, KLD decreases, and BS increases as surprise increases. Thus, in this area of surprise, maximizing both the KLD and BS at the same time is impossible. We infer that the two types of curiosity alternately maximize KLD and BS. This alternating maximization of information gains generates fluctuations of surprise. The optimal arousal level, as a maximum summation of KLD and BS, falls into this area. Therefore, the optimum arousal level, *S*_*IG*_, involves fluctuations in surprise by alternately seeking novelty and evidence, driven by the two types of curiosity.

We consider that alternating the two kinds of curiosity by increasing and decreasing prediction errors represents an ideal inquiry process that achieves optimal arousal. This process provides continuous positive emotions through the continuous acquisition of maximum information gain (i.e., epistemic value). For example, “interest” is defined as disfluency reduction in fluency–disfluency theory (Graf and Landwehr, 2015). We previously formalized disfluency reduction as free energy reduction in recognition (i.e., KLD) from increased free energy (Yanagisawa et al., 2023). This corresponds to an increase in KLD from the high-surprise state shown in Figure 9. Thus, “interest” is achieved by specific curiosity (i.e., climbing a hill of KLD from the right side in Figure 9). By contrast, increasing KLD from the low-surprise state (i.e., climbing a hill of KLD from the left side in Figure 9) may explain “pleasure” defined as an increase in fluency (Graf and Landwehr, 2015). We have previously formalized fluency as KLD in recognition (Yanagisawa et al., 2023).

BS denotes the expected information gain, as discussed in the Methods section. Active inference suggests that an agent infers an optimal policy of action that minimizes expected free energy. The expected free energy includes the negative expected information gain as an epistemic value. This epistemic value drives curious behavior (Friston et al., 2017). Thus, diversive curiosity, formalized as maximizing the BS, corresponds to curiosity in active inference. We discuss the mathematical interpretations of KLD and BS in terms of the expected free energy in a later section.

### 4.3 Effect of uncertainties on optimal arousal level and epistemic values

We analyzed the effects of prediction and observation uncertainties, manipulated using prior and likelihood variances, on optimal information gains. Table 1 summarizes the effects of the two uncertainties in four quadrants for combinations of small and large uncertainties. A small prediction variance $\sigma_{p}^{2}$ indicates that the prior belief is certain because of, for example, prior experience and knowledge. However, prior beliefs are not always correct. The prediction error represents the error of prior belief from reality. Thus, a case with small $\sigma_{p}^{2}$ and large prediction error indicates a preconceived notion. By contrast, a large $\sigma_{p}^{2}$ denotes that the prior belief is uncertain, owing to, for example, a lack of prior knowledge and experience. Thus, observation variance $\sigma_{l}^{2}$ indicates the precision of observations.

**Table 1:** Summary of the effects of likelihood variance (observation uncertainty) $\sigma_{l}^{2}$ and prior variance (prediction uncertainty) $\sigma_{p}^{2}$ on maximum information gain, max*IG*, optimal prediction errors, δ_*KLD*_, δ_*BS*_, optimal surprises, *S*_*KLD*_, *S*_*BS*_, the difference in optimal prediction errors, *D*_δ_, and difference in optimal surprises, *D*_*s*_.

$\sigma_{p}^{2}:\Rightarrow$*D*_δ_ $\sigma_{l}^{2}:\Rightarrow \max IG$, *D*_*s*_	Small $\sigma_{p}^{2}$: small *D*_δ_	Large $\sigma_{p}^{2}$:
Small $\sigma_{l}^{2}$: large max*IG* Small δ_*KLD*_ Small *S*_*KLD*_, *S*_*BS*_ large *D*_*s*_.	Small $\sigma_{l}^{2}$ and small $\sigma_{p}^{2}$: Large max*IG*, smallest δ_*KLD*_, δ_*BS*_, smallest *S*_*KLD*_, *S*_*BS*_, small *D*_δ_, large *D*_*s*_.	Small $\sigma_{l}^{2}$ and large $\sigma_{p}^{2}$: Largest max*IG*, small δ_*KLD*_, moderate δ_*BS*_, small *S*_*KLD*_, *S*_*BS*_, largest *D*_δ_, *D*_*s*_.
Large $\sigma_{l}^{2}$: small max*IG* large *S*_*KLD*_	Large $\sigma_{l}^{2}$ and small $\sigma_{p}^{2}$: smallest max*IG*, moderate δ_*KLD*_, δ_*BS*_, largest *S*_*KLD*_, moderate δ_*BS*_, smallest *D*_δ_, *D*_*s*_	Large $\sigma_{l}^{2}$ and large $\sigma_{p}^{2}$: small max*IG*, largest *S*_*KLD*_, *S*_*BS*_, large *S*_*KLD*_, largest *S*_*BS*_, moderate *D*_δ_, *D*_*s*_

*X*:⇒*Y* signifies that X dominantly affects Y. Solid and broken underlines denote positive and negative effects on epistemic emotions, respectively.

We evaluate the condition of uncertainties using four indices: maximum information gain (max*IG*), optimal prediction errors (δ_*KLD*_, δ_*BS*_), optimal surprises (*S*_*KLD*_, *S*_*BS*_), difference in optimal prediction errors (*D*_δ_), and difference in optimal surprises (*D*_*s*_). The condition with a small $\sigma_{l}^{2}$ and large *s*_*p*_ provides the largest max*IG* with the largest *D*_δ_ between small δ_*KLD*_ and moderate δ_*BS*_. A larger *D*_δ_ signifies a wider exploration range through alternations of diversive and specific curiosities. Smaller *S*_*KLD*_ and *S*_*BS*_ indicate less surprise as a cognitive load in the inquiry process. Therefore, the condition combining a small $\sigma_{l}^{2}$ and large $\sigma_{p}^{2}$ is the best solution to achieve the ideal inquiry process with the largest epistemic value (information gain; max*IG*) and the largest range of exploration (*D*_δ_) under less cognitive load (*S*_*KLD*_ and *S*_*BS*_).

The condition combining a small $\sigma_{l}^{2}$ and small $\sigma_{p}^{2}$ is expected to yield the second largest epistemic value (information gain) under less cognitive load (*S*_*KLD*_, *S*_*BS*_); however, the range of exploration (*D*_δ_) is small. The condition with a large $\sigma_{l}^{2}$ and large $\sigma_{p}^{2}$ is expected to result in a small information gain with a moderate range of exploration at the largest prediction error level. The condition combining a large $\sigma_{l}^{2}$ and small $\sigma_{p}^{2}$ is the worst case, corresponding to the smallest information gain and the smallest exploration range.

As an overall trend, an increase in prediction variance $\sigma_{p}^{2}$ expands the range of exploration (*D*_δ_). This suggests that highly certain prior beliefs, such as preconceived notions or strong assumptions, tend to suppress the range of exploration, whereas an open mind involving a flat prior belief facilitates a range broader of exploration. The observation variance $\sigma_{l}^{2}$ decreases the expected maximum information gain (max *IG*). This suggests that precise observation increases expected information gains (epistemic value) with positive emotions. $\sigma_{l}^{2}$ can be decreased in different ways, for example, by increasing the precision of stimuli, paying attention to stimuli, and improving the accuracy of the observation models.

### 4.4 Expected free energy and information gains in action

Free energy before updating belief is decomposed into a summation of KLD and surprise. Furthermore, surprise (−ln*p*(*o*)) is factorized into BS and inverse accuracy:


(19) $$\begin{array}{c} F={\langle \ln q\left(s\right)-\ln p\left(s,o\right)\rangle }_{q\left(s\right)}=D_{KL}\left[q\left(s\right)\|p\left(s|o\right)\right]-\ln p\left(o\right)\\ =D_{KL}\left[q\left(s\right)\|p\left(s|o\right)\right]+D_{KL}\left[p\left(s|o\right)\|p\left(s\right)\right]-{\langle \ln p\left(o|s\right)\rangle }_{p\left(s|o\right)}. \end{array}$$

**Figure 10:**
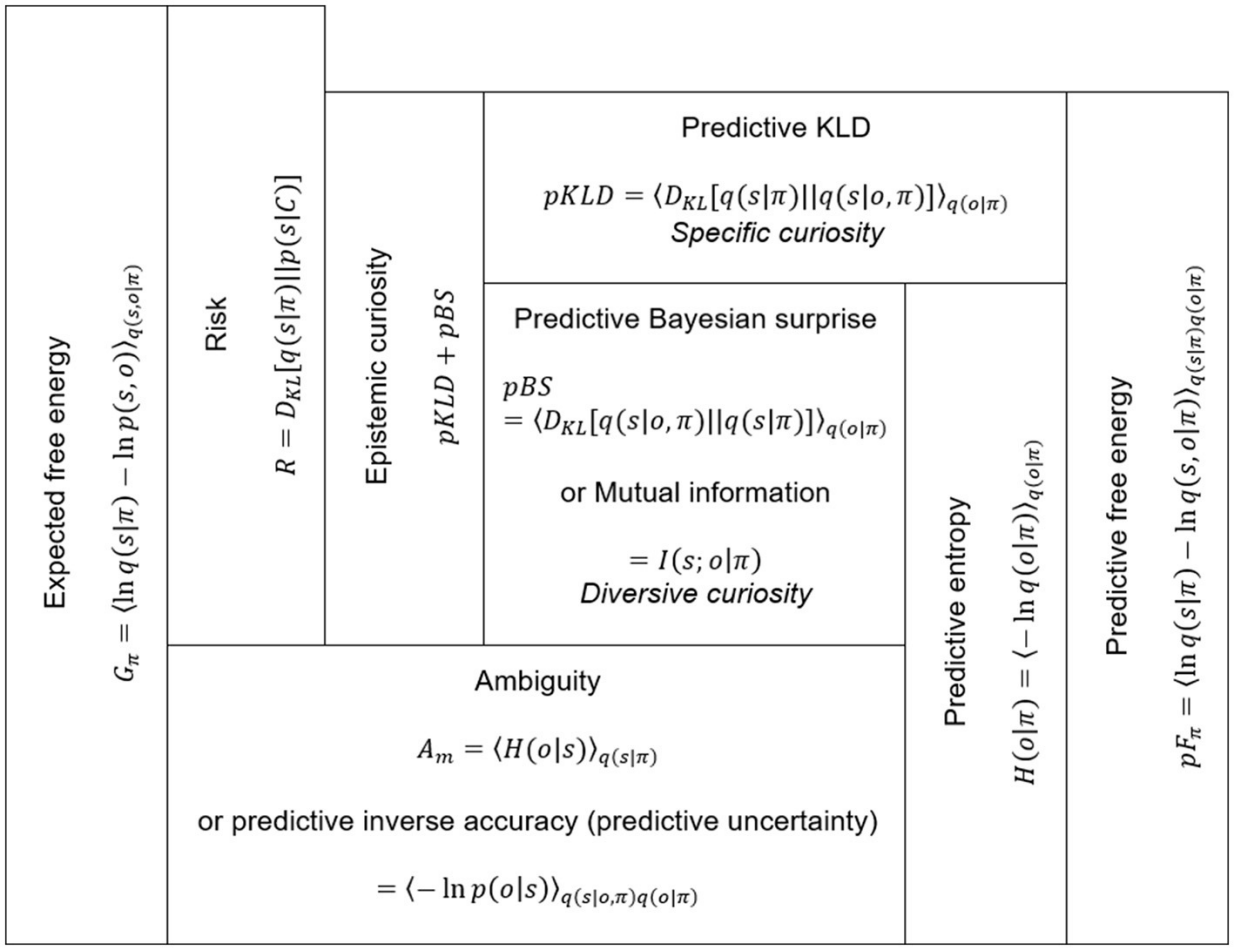
Expected and predictive free energy and their decompositions. Epistemic curiosity in action may link to the two predictive information gains, pKLD and pBS. These predictive information gains decrease ambiguity, which is equivalent to non-risk expected free energy.

Consider predictive free energy of a predictive distribution *q*(*o*|π) given by future action under a policy π as free energy averaged over the predictive distribution.


(20) $$\begin{array}{c} pF_{\pi }={\langle \ln q\left(s|\pi \right)-\ln q\left(s,o|\pi \right)\rangle }_{q\left(s|\pi \right)q\left(o|\pi \right)} \end{array}$$

where *q*(*s, o*|π) consists of a product of a prior belief under a policy and a likelihood, *q*(*s*|π)*p*(*o*|*s*). The predictive observation is defined as a marginal likelihood $q\left(o|\pi \right)=\int_{-\infty }^{\infty }q\left(s|\pi \right)p\left(o|s\right)ds$. By applying the two-step decompositions of Equations 19, 20, we obtain the following two formulas (also see Figure 10).


(21) $$\begin{array}{l} pF_{\pi }={\langle D_{KL}[q(s|\pi )||q(s|o,\pi )]-\ln q\left(o|\pi \right)\rangle }_{q\left(o|\pi \right)}\\ \;=\langle D_{KL}\left[q(s|\pi )||q\left(s|o,\pi \right)\right]+D_{KL}\left[q(s|o,\pi )||q\left(s|\pi \right)\right]\\ \;-{{\langle \ln p\left(o|s\right)\rangle }_{q\left(s|o,\pi \right)}\rangle }_{q\left(o|\pi \right)} \end{array}$$

The first line of Equation 21 is a summation of predictive KLD (pKLD) and predictive entropy, *H*(*o*|π). The first term, pKLD, indicates the information gain of a predictive belief update averaged over predictive distribution. pKLD is the expected information gain when recognizing predictive observations given under a policy. The second term, predictive entropy, is decomposed into predictive BS (pBS) and ambiguity (Am). pBS equals the mutual information of states and observations under a policy.


(22) $$\begin{array}{c} pBS={\langle D_{KL}\left[q\left(s|o,\pi \right)\|q\left(s|\pi \right)\right]\rangle }_{q\left(o|\pi \right)}\\ ={\langle \ln q\left(s,o|\pi \right)-\ln q\left(s|\pi \right)q\left(o|\pi \right)\rangle }_{q\left(s,o|\pi \right)}=I\left(s;o|\pi \right) \end{array}$$

Mutual information indicates a measure of interdependence between states and observations. The greater the mutual information, the more precise the generative model's knowledge of the relationships between states and observations. Hence, pBS represents predictive information gain by the expected learning of the relationship between states and observations. By applying the link between the two types of curiosities and KLD and BS in recognition, we consider that the pKLD and pBS correspond to specific and diversive curiosities in action, respectively, and the summation of pKLD and pBS is thought to represent epistemic curiosity as a drive to minimizing predictive free energy.

Ambiguity is the information potential that is expected to remain after the recognition and learning process regarding the predictive observations under a policy. Ambiguity equals inverse predictive accuracy. It indicates an expected likelihood of entropy under a policy.


(23) $$\begin{array}{c} {Amgibuity=\langle -\ln p\left(o|s\right)\rangle }_{q\left(s,o,\pi \right)}={\langle H\left(o|s\right)\rangle }_{q\left(s|\pi \right)} \end{array}$$

An active inference framework suggests that an agent's action policy is selected so as to minimize expected free energy (Friston et al., 2017; Parr et al., 2022; Smith et al., 2022). The expected free energy is reshaped to the summation of risk and ambiguity.


(24) $$\begin{array}{c} G_{\pi }={\langle \ln q\left(s|\pi \right)-\ln p\left(s,o|\pi \right)\rangle }_{q\left(s,o|\pi \right)}=risk+ambiguity \end{array}$$

(25) $$\begin{array}{c} risk=D_{KL}\left[q\left(o|\pi \right)\|p\left(o|C\right)\right] \end{array}$$

where *p*(*o*|*C*) is a desired observation distribution called preference. It is an observation prior as a component of a generative model, i.e., *p*(*s, o*|π) = *p*(*o*|*C*)*q*(*s*|*o*, π). When predictive distinction approximates the preference, *q*(*o*|π) ≈ *p*(*o*|*C*), the risk becomes zero, and thus expected free energy approximates ambiguity. In this case, the summation of pKLD and pBS indicates the potential difference from predictive free energy to expected free energy without risk or ambiguity. As the expected information gain increases by recognition and learning processes about the states given predictive observation under certain policies, the potential gap increases, and as a result, ambiguity decreases. Therefore, selecting a curious action policy that is expected to maximize the predictive information gains is likely to minimize ambiguity (uncertainty about relations of states and observations). This corresponds to active inference without risk or preference. Such an agent, like an ideal researcher, would act solely out of epistemic curiosity.

### 4.5 Limitations and further discussions

The analytical results are based on a Gaussian generative model. A Gaussian model was used to independently manipulate the prediction errors and uncertainties and to analyze their effects on information gains. Although the Laplace approximation and the principle of maximum entropy reasonably support the Gaussian assumption, true distributions can be more complex than Gaussian distributions. For specific applications with complex distributions, further analysis is based on the method proposed in this study.

This study focuses on emotions induced by epistemic values (epistemic emotions), such as curiosity and interest. However, emotions are affected by individual preferences and appraisals of the situation against objectives (Ellsworth and Scherer, 2003). We may expand the emotion model to include such preference-based emotions by introducing the pragmatic value formalized as a risk term in expected free energy (Parr et al., 2022). The model does not consider the individual capacity to process information. Surprise (free energy) exceeding the capacity may lead to negative emotions.

This study was limited to analyzing two types of information gain linked to epistemic emotions as functions of surprise in a context-independent manner. Epistemic emotions based on epistemic values, such as curiosity, can be observed through the agent's behavior. Active inference, where an action policy is inferred to minimize expected free energy, can be used to simulate agent behavior based on epistemic emotions in a specific context (Friston et al., 2017). As discussed, the expected free energy comprises two types of information gain. In future studies, it will be necessary to accumulate evidence of the model predictions based on correspondence between agent simulations and actual human behavior across various specific contexts.

## 5 Conclusion

This study mathematically formulates the arousal potential functions of epistemic emotions, such as curiosity and interest, that drive inquiry processes based on information gains. Decrements in free energy in Bayesian recognition and prior belief updates correspond to two types of information gain, i.e., KLD and BS, respectively. Free energy reduction induces positive emotions by diminishing the surprise caused by prediction errors and uncertainty, which provide information gains (i.e., epistemic value). We demonstrate that the two types of information gain form upward-convex curve functions of surprise using a Gaussian generative model with a uniform noise likelihood and define epistemic emotions as information gains (or decrements of free energy). An exhaustive analysis using the model reveals the effects of prediction and observation uncertainties on the peak of information gain functions as the optimal arousal level. Specifically, the analytical results suggest that the greater the prediction uncertainty and the lower the observation uncertainty, the greater the information gained through a larger exploration range.

These results provide broad and fundamental insights into enhancing the valence of epistemic emotions that facilitate the inquiry process. This model is derived from the synthesis of free energy minimization, proposed as the first principle of brain function, and the well-established arousal potential theory. As such, this modeling framework is applicable across various domains concerned with epistemic emotions and motivation, including education, creativity, aesthetics, affective computing, and related areas within the cognitive sciences. Further studies are needed to empirically validate this principle-based model and to deepen our understanding of the relationship between the inquiry process and emotional dynamics in diverse and complex situations.

## Data Availability

Publicly available datasets were analyzed in this study. This data can be found at: https://osf.io/6xcaz/.

## Appendix

We show the results of the KLD and BS calculations in Section 3.2 when the likelihood function in the form of a uniform distribution added to a normal distribution (*p*_ε_(*o*|*s*)) is used. Using the posterior function (*p*_ε_(*s*|*o*)), we derived KLD and BS:

(26)
$$\begin{array}{c} KLD=D_{KL}\left[p\left(s\right)\|p_{\varepsilon }\left(s|o\right)\right]=KLD_{N}+\ln\left(1+\frac{\varepsilon }{e\left(\delta \right)}\right)-I,\; \end{array}$$

where *KLD*_*N*_ is a KLD using only the Gaussian likelihood (see Equation 14), and *I* is an improper integral:

(27)
$$\begin{array}{c} I=\int_{-\infty }^{\infty }N_{pri}\ln\left(1+\frac{\varepsilon N_{pri}}{e\left(\delta \right)N_{post}}\right)ds\\ =\int_{-\infty }^{\infty }N_{Pri}\ln\left[1+\varepsilon \sqrt{2\pi }\sigma_{l}\;\exp\left\{\frac{{\left(s-o\right)}^{2}}{2\sigma_{l}^{2}}\right\}\right]ds. \end{array}$$

Using the KLD, we derived BS as follows:

(28)
$$\begin{array}{c} BS=D_{KL}\left[p_{\varepsilon }\left(s|o\right)\|p\left(s\right)\right]\\ =\frac{1}{e\left(\delta \right)+\varepsilon }\left[e\left(\delta \right)\left\{BS_{N}-\ln\left(1+\frac{\varepsilon }{e\left(\delta \right)}\right)+J\right\}-\varepsilon KLD\right], \end{array}$$

where *BS*_*N*_ is the *BS* of using only the Gaussian likelihood (see Equation 15), and *J* is an improper integral:

(29)
$$\begin{array}{c} J=\int_{-\infty }^{\infty }N_{post}\ln\left(1+\frac{\varepsilon N_{pri}}{e\left(\delta \right)N_{post}}\right)ds\\ =\int_{-\infty }^{\infty }N_{post}\ln\left[1+\varepsilon \sqrt{2\pi }\sigma_{l}\;\exp\left\{\frac{{\left(s-o\right)}^{2}}{2\sigma_{l}^{2}}\right\}\right]ds. \end{array}$$
